# Prediction of protein continuum secondary structure with probabilistic models based on NMR solved structures

**DOI:** 10.1186/1471-2105-7-68

**Published:** 2006-02-14

**Authors:** Mikael Bodén, Zheng Yuan, Timothy L Bailey

**Affiliations:** 1 School of Information Technology and Electrical Engineering, The University of Queensland, QLD 4072, St Lucia, Australia; 2 Institute of Molecular Bioscience, The University of Queensland, QLD 4072, St Lucia, Australia

## Abstract

**Background:**

The structure of proteins may change as a result of the inherent flexibility of some protein regions. We develop and explore probabilistic machine learning methods for predicting a continuum secondary structure, i.e. assigning probabilities to the conformational states of a residue. We train our methods using data derived from high-quality NMR models.

**Results:**

Several probabilistic models not only successfully estimate the continuum secondary structure, but also provide a categorical output on par with models directly trained on categorical data. Importantly, models trained on the continuum secondary structure are also better than their categorical counterparts at identifying the conformational state for structurally ambivalent residues.

**Conclusion:**

Cascaded probabilistic neural networks trained on the continuum secondary structure exhibit better accuracy in structurally ambivalent regions of proteins, while sustaining an overall classification accuracy on par with standard, categorical prediction methods.

## Background

Protein structures can be characterized by regular folding patterns. Descriptions at the level of local folding pattern (e.g., alpha helix or beta sheet) are known as the protein's *secondary *structure, as opposed to its full *tertiary *(3-dimensional) structure. It is common practise to describe each residue as belonging to either one of eight secondary structure environment classes:

*C*_8 _= {*G*, *H*, *I*, *E*, *B*, *T*, *S*, *C*},

or to one of three classes:

*C*_3 _= {*H*, *E*, *C*}.

The set *C*_8 _consists of the eight DSSP [1] classes: 3_10_-helix (*G*), alpha helix (*H*), pi helix (*I*), helix-turn (*T*), extended beta sheet (*E*), beta bridge (*B*), bend (*S*) and other/loop (*C*). In set *C*_3_, class *H *contains the 3_10_-helix and alpha helix classes, class *E *contains the extended beta sheet and beta bridge classes and class *C *contains the remaining four DSSP classes.

In addition to the dominating covalent polypeptide backbone, the stability of a protein structure is determined by the collective strength of many covalent and ionic bonds, as well as van der Waals attractions. However, it is well established that protein structures are not entirely rigid. As the tertiary structure of a protein changes due to thermal motion or outside in influences, a residue may also change secondary structure states. In stark contrast to the typical definition of secondary structures in which a residue can only have a single state, *continuum *secondary structures allow a residue to be in all states, indicated by a probability distribution over the possible secondary structure states. It has been contended that specifying protein structure this way allows regions of transition in secondary structure (e.g., caps) to be characterised more accurately [2]. Moreover, a probabilistic representation of secondary structures sheds light on the conformational flexibility of proteins [2,3].

Solved by X-ray crystallography, a protein has conformational variation mainly due to different experimental conditions. On the other hand, NMR solution of a protein structure always provides a number of models with structural variation due, at least in part, to *intrinsic motions of the protein *[2]. Andersen and colleagues developed a scheme-DSSPCONT-where the secondary state probability distribution for each residue in a protein is estimated from the variation amongst an ensemble of NMR models of the protein. The DSSPCONT assignment thus directly distinguishes between less flexible regions and more flexible ones [2].

In this work, we use the same NMR dataset as Anderson *et al*. [2] to develop probabilistic models that are able to predict the continuum secondary structure from the amino acid sequence. We test these models using a dataset of continuum secondary structures developed by us and having very low sequence similarity with the Anderson *et al*. dataset. Given a target protein sequence, for each residue, our models are trained to predict the *probability distribution *over all possible secondary structure environments for that residue. Importantly, the probabilistic models are thus directly provided with prediction targets that reflect the variability of conformation.

There are a large number of prediction methods that take as input a protein sequence and predict the secondary structure of each of its residues. Current best methods (including PSIPRED, SSPro, PROFsec and others) achieve a 3-class accuracy (*Q*_3_) of 75–80% [4-11]. These and other previous secondary structure prediction methods implicitly assume that each residue in the protein belongs to a definite secondary structure. The target secondary structure used by most models are categorically determined by DSSP [1] or STRIDE [12].

Most previous prediction methods provide continuous (rather than categorical) outputs, and it is tempting to interpret these as probabilities. What distinguishes our approach is that we train our models with probabilistic data, so it is entirely natural to interpret their predictions as probabilities. Previous approaches train models using categorical data, so non-categorical outputs often do not represent probabilities at all. In most cases (e.g., with neural nets trained on categorical data), non-categorical outputs represent the distance from an internal decision boundary, which may be correlated with the certainty of the prediction, but is not a probability in the strict sense of the word. It is also unclear whether such outputs bear direct physical or biological meanings (like thermal motion or conformational switching), or if they merely reflect the confidence that the model has in the prediction. Moreover, it is estimated that 5–15% of the current prediction errors can be attributed to the current rigid definition of secondary structure and how it is derived from experimental models [13].

In this work, we study three models of increasing complexity: Naive Bayes' Density Predictors (NBDP), Probabilistic Neural Networks (PNN), and Cascaded Probabilistic Neural Networks (CPNN). The first of these methods (NBDP) is the simplest to implement, but makes the most assumptions about the data. In particular, NBDP assumes that the identities of adjacent residues in the protein are independent given the secondary structure. This is obviously not true in general, but we include results using NBDP because it is often a surprisingly effective method for learning probability distributions [14]. The neural network models are known to be effective for categorical secondary structure prediction [4,6,15] and are thus explored here, too.

To quantify the accuracy of our models, we measure the divergence between the probabilities derived from high-quality NMR models and the predicted probabilities. In combination with each of the three model types, we also examine the effect of describing the amino acids in the input sequence in two different ways. We refer to the two residue description methods as the *amino acid identity *and PSI-BLAST *profile *methods, where the latter is employed in the top performing categorical secondary structure predictors.

Our main concern is to establish how well the continuity of structure can be captured by machine learning models from limited datasets. We are specifically interested to see if secondary structure prediction can be improved by training the model with the fine-grained structural data from NMR.

To compare our work with previous studies, we also convert the continuum predictions made by our models into categorical predictions by selecting the class with the highest predicted probability. We compare these results to those of a categorical prediction method trained on the data obtained by a similar conversion of the continuum secondary structure data to categorical. The categorical method we compare to is a Cascaded Categorical Neural Network (CCNN), as employed by the top-performing PSIPRED [4] algorithm.

## Results

The probabilistic models studied here are more accurate at predicting continuum secondary structure (residue class density) than models trained on categorical data. The difference in accuracy is most pronounced for residues with structural ambivalence. Furthermore, these probabilistic models can be used to predict categorical secondary (residue class) with accuracy comparable to a successful categorical method. These results hold when accuracy is measured by cross-validation on the training data as well as when validated with an test dataset containing only proteins with low sequence similarity to proteins in training set, and for both the 3- and 8-class prediction problems.

### Probabilistic models

The PNN and CPNN are the most successful models at predicting continuum secondary structure in our study. Accuracy is highest when the residues in the sequence are described using the PSI-BLAST profile method. The accuracy of the PNN and CPNN methods is also sensitive to the number of hidden nodes in the model and to the width of the sequence window presented to the model. The accuracy of the Naive Bayes model is substantially inferior to the that of the PNN and CPNN models.

We use the Kullback-Leibler (KL) divergence to measure the accuracy of our models at predicting continuum secondary structure (see Methods section). Accuracy increases with *decreasing *KL divergence. The predictive accuracy of the PNN and CPNN models generally improves as the number of hidden nodes increases (Table 1), although improvement is slight beyond 25 hidden nodes. The optimal window size is 15 residues for both the 3- and 8-class prediction problems. The KL divergence of the best PNNs is 0.49 and 0.88 for the 3- and 8-class problems, respectively. The CPNN improves this accuracy to 0.47 and 0.84, respectively.

**Table 1:** Cross-validated density prediction accuracy of PNN and CPNN models. Average KL divergence for probabilistic and cascaded probabilistic neural network models predicting continuum 3- and 8-class secondary structure from PSI-BlAST encoded sequence data. Window size and number of hidden nodes are varied. All predictions are for 10-fold cross-validation on the training set (set-174). When standard errors are given in parentheses, the predicted value is the mean of five randomized repeats of cross-validation. in parenthesis.

*number of classes*	*window size*	*PNN hidden nodes*							*CPNN hidden nodes*
		0	5	10	15	20	25	30	30
3	11	0.59	0.57	0.53	0.52	0.52	0.52	0.51	
	13	0.58	0.57	0.53	0.52	0.51	0.51	0.51	
	15	0.58	0.56	0.53	0.52	0.51	0.50	0.49 (0.002)	0.47 (0.002)
	17	0.58	0.56	0.54	0.52	0.51	0.51	0.51	
	19	0.59	0.56	0.54	0.52	0.51	0.52	0.51	

8	11	0.97	0.97	0.94	0.91	0.92	0.89	0.90	
	13	0.97	0.96	0.92	0.90	0.90	0.89	0.89	
	15	0.97	0.95	0.92	0.90	0.90	0.89	0.88 (0.001)	0.84 (0.002)
	17	0.98	0.96	0.93	0.91	0.90	0.89	0.89	
	19	0.98	0.97	0.94	0.91	0.92	0.89	0.90	

For the 3-class problem, the best Naive Bayes' model (with an 11-residue window) achieves a KL divergence of 0.74. For the 8-class problem, KL divergence is 1.19 for a model with a 7- and 9-residue window. The NBDP model is far inferior to the other models when residues are described using the amino acid identity method (Table 2), and fails almost totally with the PSI-BLAST profile description method (data not shown). The optimal window size for NBDP, of approximately 10 residues, is smaller than for the PNN model.

**Table 2:** Cross-validated density prediction accuracy of NBDP models. Average KL divergence for the Naive Bayes' Density Predictor is shown for the 3- and 8-class prediction tasks with varying sequence window sizes. The residues in the window were described using the amino acid identity method. All predictions are for 10-fold cross-validation on the training set (set-174). Best results are shown in bold.

*window size*	*3-class accuracy*	*8-class accuracy*
5	0.77	1.20
7	0.75	**1.19**
9	0.75	**1.19**
11	**0.74**	1.20
13	0.75	1.22
15	0.76	1.25
17	0.78	1.28
19	0.79	1.31

**Figure 1:**
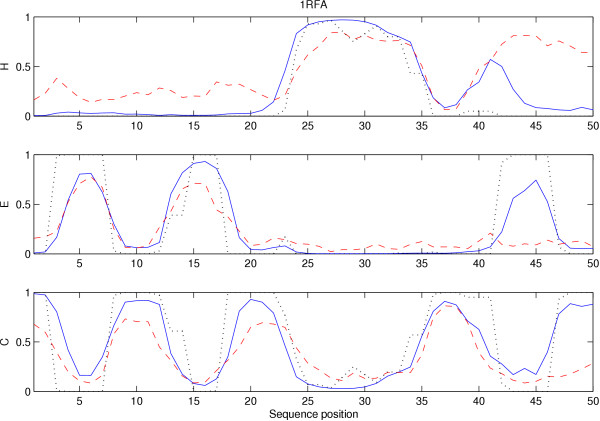
**Example 3-class continuum secondary structure predictions**. The 3-class predictions of the best NBDP and CPNN models for positions 1–50 of protein PDB:1RFA are plotted. The target (known) probabilities are plotted as a dotted black line. The dashed red line is the NBDP predictions and the solid blue line is the CPNN predictions.

To give the reader a better qualitative feeling for the meaning of various KL divergences, we show the output of the most accurate 3-class NBDP and CPNN models on the Ras-binding domain of C-Raf-1 (PDB:1RFA) in Figure 1. The average KL divergence of the NBDP prediction on this sequence is 0.67, and is slightly worse than the average 3-class prediction accuracy for the PNN model (see Table 1), and about average for the NBDP model (see Table 2). The average divergence of the CPNN prediction for this sequence 0.27, slightly more accurate than the overall average achieved by the CPNN model on all test sequences (see Table 1). The data in the figure is for NBDP using the amino acid identity residue description method, and for CPNN using the PSI-BLAST profile method.

### Comparing with categorical models

Even though our probabilistic models are not explicitly trained to produce categorical output, they perform competitively with a state-of-the-art classification method. We train Cascaded Categorical Neural Networks (CCNNs) (similar to PSIPRED) to predict the categorical targets using a configuration identical to the best CPNN in this study (30 hidden nodes, 15-residue window). These CCNNs are trained using categorical data derived from the continuum data, as described in the Methods section.

The classification accuracy of the probabilistic model (CPNN) is comparable to that of the categorical model (CCNN) in the 3-class problem using several popular accuracy metrics including *Q*_3 _and SOV (Table 3). We observe that, with a *Q*_3 _of 77.3, the CPNN is on par with the CCNN (*Q*_3 _= 77.2). (*Q*_3 _measures classificaton accuracy on a scale of 0 (worst) to 100 (best).) Similarly, the two models have segment overlap-based SOV measures [16] of 73.8 (CPNN) and 72.8 (CCNN). (SOV measures a segment-based precision of prediction ranging from 0 (worst) to 100 (best).) Compared with the cascaded probabilisitic model (CPNN), the PNN model has similar *Q*_3 _accuracy but notably inferior SOV. The best Naive Bayes' Density Predictors with Boolean input features manage a *Q*_3 _of 61.2 and an SOV of 52.9, significantly worse than the other probabilistic models.

**Table 3:** Cross-validated classification accuracy of all models. Average accuracy of categorical prediction in the 3- and 8-class problems is given as measured by the accuracy metric *Q*_*k*_, the Matthews correlation, *r*(), and SOV. All predictions are for 10-fold cross-validation on the training set (set-174). When standard errors are given in parentheses, the predicted value is the mean of five randomized repeats of cross-validation. The best results are shown in bold.

*model*	*3-class problem*					*8-class problem*
	
	*Q*_3_	*r*(*H*)	*r*(*E*)	*r*(*C*)	SOV	*Q*_8_
NBDP	61.2	0.40	0.34	0.41	52.9	46.1
PNN	76.4 (0.09)	0.68	0.62	0.57	67.4	61.4 (0.09)
CPNN	**77.3 **(0.07)	**0.69**	**0.63**	0.58	**73.8**	**62.8 **(0.05)
CCNN	77.2 (0.08)	**0.69**	**0.63**	**0.59**	72.8	62.5 (0.15)

As an independent test, we also test the models on the all sequences in the small CAFASP3 data used to benchmark a range of public predictors [17]. None of the sequences in CAFASP3 are included in our training set (set-174). We find that both CPNN and CCNN achieve a *Q*_3 _of 76.2, only slightly worse than their accuracies measured by cross-validation on the training set (Table 3). For comparison, *Q*_3 _accuracies reported for other categorical models in the Eyrich study [17] ranged from 67.5 to 78.9 (78.6 for PSIPRED). Similarly, the CPNN and CCNN models have SOV measures of 73.5 and 73.9, respectively, which are slightly better than their cross-validated accuracies. The classification accuracy of the CPNN is slightly lower than the best results reported in the literature, but this is to be expected because our training set is considerably smaller than those used in many previous studies [7]. It also bears noting that the probabilistic model (CPNN) is not specifically trained to produce categorical targets.

Conversely, many models trained on categorical data also offer continuous predictions. For the CCNN fitted with the softmax output function, we can evaluate its ability to directly produce an output close to the continuum secondary structure. On the 3-class problem, the CCNN achieves KL divergence (averaged over all residues) that is nearly identical to that of the CPNN (Table 4, columns labeled "entropy ≥ 0.0"). The cross-validated KL divergence on all residues is 0.48 (SE = 0.002) for the CPNN, which is close to that of CCNN (0.47). Likewise, the CCNN and CPNN have very similar KL divergence on the test dataset (test-286): 0.51 and 0.50, respectively. On the 8-class problem, the CCNN and CPNN have very similar overall KL divergence on the test dataset (0.99 vs 0.98), but CCNN appears slightly inferior when cross-validated on the training set: 0.87 (SE = 0.003) and 0.84, respectively. We note that the categorical model (CCNN) is not specifically trained to produce continuous targets.

**Table 4:** Density prediction accuracy (KL divergence) for structurally ambivalent residues. Average KL divergence of prediction of continuum secondary structure for residues that have a structural ambivalence equal to or exceeding an entropy of 0.0 (all residues), 0.3 and 0.5. "CV": average (standard error) of five randomized repeats of 10-fold cross-validation on the training set (set-174). "test": average error on the test dataset (set-286).

*problem*	*model*	*entropy *≥ 0.0		*entropy *≥ 0.3		*entropy *≥ 0.5	
		
		*CV*	*test*	*CV*	*test*	*CV*	*test*
3-class	PNN	0.49 (0.002)	0.52	0.53 (0.002)	0.59	0.52 (0.003)	0.54
	CPNN	0.47 (0.002)	0.50	0.53 (0.003)	0.57	0.53 (0.003)	0.53
	CCNN	0.48 (0.002)	0.51	0.58 (0.002)	0.62	0.59 (0.004)	0.58

8-class	PNN	0.88 (0.001)	1.01	1.07 (0.003)	1.26	1.07 (0.004)	1.13
	CPNN	0.84 (0.002)	0.98	1.03 (0.004)	1.22	0.98 (0.008)	1.15
	CCNN	0.87 (0.003)	0.99	1.12 (0.004)	1.31	1.10 (0.010)	1.24

To investigate the qualitative difference between models that are trained on probabilistic targets and categorical targets, we focus on residues in "fuzzy" regions. In particular, we identified "fuzzy" residues as those with an observed secondary structure state entropy of 0.3 or above (15% of all residues), and "very fuzzy" residues as those with target entropy of 0.5 or above (8% of all residues). We investigate the accuracy of the models created by cross-validation on the full training set on each of these low entropy residue subsets (Tables 4 and 5).

**Table 5:** Classification accuracy (*Q*_3_) for structurally ambivalent residues. Average accuracy as measured by *Q*_3 _of 3-class categorical prediction of residues that have a structural ambivalence equal to or exceeding an entropy of 0.0 (all residues), 0.3 and 0.5. "CV": average (standard error) of five randomized repeats of 10-fold cross-validation on the training set (set-174). "test": average error on the test dataset (set-286).

*problem*	*model*	*entropy *≥ 0.0		*entropy *≥ 0.3		*entropy *≥ 0.5	
		
		*CV*	*test*	*CV*	*test*	*CV*	*test*
3-class	PNN	76.4 (0.09)	77.0	55.5 (0.18)	50.3	50.2 (0.18)	49.0
	CPNN	77.3 (0.07)	77.8	55.7 (0.22)	50.6	50.4 (0.24)	49.0
	CCNN	77.2 (0.08)	77.8	55.4 (0.17)	51.5	50.2 (0.31)	50.0

**Figure 2:**
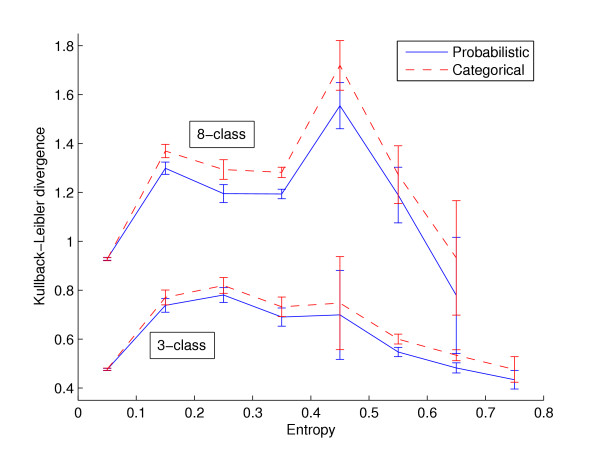
**KL divergence as a function of test dataset residue entropy**. The dashed red and solid blue lines show the KL divergence of predictions on the test dataset (set-286) made by the CCNN and CPNN models, respectively. Residues are binned by secondary structure entropy, and the mean KL divergence of residues in a bin is plotted at the midpoint of the bin. Error bars show plus and minus one standard error around each mean. The numbers of residues in the bins for the 3-class problem are (in order of increasing entropy) 26357, 948, 839, 525, 14, 1036, 489, 4 and 2. For the 8-class problem bin occupancies are 25657, 1878, 777, 1728, 127, 43, 4 and 0.

Both probabilistic models (PNN and CPNN) have significantly lower KL divergence on residues with structural entropy greater than 0.3 when compared with the categorical model (CCNN). This conclusion holds when KL divergence is measured using cross-validation on the training set as well as when measured on the test dataset (Table 4, last four columns). The probabilistic models have significantly superior (lower) KL divergence than the categorical model for both the 3- and 8-class problems. For example, the cross-validated 3-class KL divergence for residues with entropy at least 0.5 is 0.59 for the CCNN, but only 0.53 for the CCNN. Similarly, the average 3-class KL divergence on the test dataset residues with entropy at least 0.5 is, 0.58 and 0.53, respectively for the CCNN and CPNN models. The best probabilistic model (CPNN) is superior to the best categorical model (CPNN) for predicting continuum secondary structure for test dataset residues with entropy greater than 0.1 (Figure 2). This result holds for both the 3- and 8-class problems.

For the 3-class problem, KL divergence on the test dataset is only slightly higher than the cross-validated value (Table 4). This shows that overfitting is not a serious problem with the 3-class models. On the other hand, the 8-class models show significantly worse KL divergence on the test dataset than during cross-validation. This may be caused by overfitting given the small size of the training dataset (approximately 17000 residues) compared to the number of parameters in the models (approximately 9000). However, since overfitting does not occur with the (equally complex) 3-class models, it is likely that the more complex output space in the 8-class problem is the true culprit.

For *classifying *structurally ambivalent residues (Table 5, last four columns), the probabilistic networks (PNN and CPNN) are on par with the categorical neural network (CCNN).

Three-class classification accuracy (*Q*_3_) on residues with entropy at least 0.3 is 55.7% (SE = 0.22) and 55.4% (SE = 0.17) for the CPNN and CCNN models, respectively It is worth noting that the probabilistic networks achieve good classification despite not being specifically trained for this task.

## Discussion

Our results support the existence of higher-order dependencies between the residues within the input window as the Naive Bayes' models and single-layer Probabilistic Neural Networks perform relatively poorly.

In agreement with previous work on both neural networks and support vector machines [4], we note that the PSI-BLAST profile description of the sequence data works much better than the amino acid identity method for all types of Probabilistic Neural Networks. However, the opposite holds for the Naive Bayes' Density Predictor. With the PSI-BLAST profile description method, the performance of NBDP drops considerably. On closer inspection, the class conditioned distributions of input values are strongly overlapping and consequently result in poor discrimination.

To investigate whether the input values follow a non-Gaussian distribution we also tried dividing each numeric input value into 5 and 10 bins. The performance of the Naive Bayes' Density Predictor then drops even further. The naive assumption of independence among the very large number of random variables (resulting from the profile description method) is clearly violated. Each residue profile column reflects a single piece of information involving all of the twenty amino acids, and the random variables making up the column are highly dependent. This fact is most likely responsible for the failure of NBDP with the PSI-BLAST profile residue description method.

Even though the *average *KL divergence between the probabilistic target and predicted values are almost the same for both the CCNN and CPNN, they seem to handle structurally ambivalent residues differently. The discrepancy as measured on these challenge subsets indicates that training with continuum data results in more accurate prediction for residues with high structural ambivalence.

Our simulations indicate that it is not sufficient to train on the categorical targets if structurally ambivalent states need to be characterised precisely. This precision may be particularly important for identifying conformational flexibility, e.g. Young *et al*. [18] rely on a reliability index of a categorical prediction to identify conformational switches.

## Conclusion

The models we present are adapted to predict a continuum secondary structure, i.e. to predict the probability of a residue belonging to any of the three or eight secondary structure classes. The probabilities derive from NMR models that capture some aspects of protein flexibility-in contrast to most categorical predictors which are trained on categorical data usually derived from X-ray crystal structures.

Cascaded Probabilistic Neural Networks using a 15-residue input window (involving primary sequence data only) are able to produce 3-class predictions that, on average, measure 0.47 using the Kullback-Leibler divergence from target distributions. A similar model produces 8-class secondary structure predictions measuring an average KL divergence of 0.84 from the observed targets.

To illustrate the performance and utility of probabilistic models, we also convert the probabilistic predictions to categorical classifications and note that the probabilistic models are on par with models that are directly trained on categorical data. In particular, structurally ambivalent residues (e.g. caps of regular folds) are predicted more accurately by the best probabilistic models than by their categorical counterparts. So far, the scarcity of NMR data renders the continuum secondary structure prediction less accurate for classification than categorical models directly trained on much larger sets of crystallographic data.

## Methods

### Overview

A typical machine learning approach might view the secondary structure environment of a protein residue as a *multinomial random variable*, *C*, having *k *possible values. Our view is that the secondary structure environment of a residue does not have a single, fixed value, but may be in any one of *k *classes. The occupation of the structural classes follows a multinomial distribution, and is measured by the variation among NMR models. We start from a training set, *S*, of examples each of the form *E *=*<***X**, **T ***>*. The goal is to use the training set to learn a function **Y **= *f*(**X**) that approximates the posterior probabilities, **T **=<*T*_1_, *T*_2_, ..., *T*_*k *_>, where

*T*_*j *_= *Pr*(*C *= *j*|**X**), 1 ≤ *j *≤ *k*.

In common with many earlier secondary structure prediction methods, we use a *sliding window approach*. That is, the prediction for a particular residue will be based on a description (see below) of that residue and some number of residues on either side of it in the protein sequence. The sequence window,

**X **=<*X*_1_, *X*_2_, ..., *X*_*w*-1_, *X*_*w *_>,

is used to describe the residue at the center of a *w*-residue window. The entry *X*_*i *_in this vector is a description of the residue at the *i*th position (along the sequence) in the window.

Our approach outputs a *probability vector*,

**Y **=<*Y*_1_, ..., *Y*_*k *_>

in the *k*-class problem, where *Y*_*j *_is the probability that the central residue in sequence window **X **is in the *j*th environment of *C*_3 _(3-class problem) or *C*_8 _(8-class problem). (For convenience, we use integers to refer to the environment classes, substituting the integer *j *for the *j*th entry in either the 3- or 8-class sets.) Since **Y **is a probability vector, we have the constraints:

0 ≤ *Y*_*j *_≤ 1, 1 ≤ *j *≤ *k*,

and

∑j=1kYj=1.
 MathType@MTEF@5@5@+=feaafiart1ev1aaatCvAUfKttLearuWrP9MDH5MBPbIqV92AaeXatLxBI9gBaebbnrfifHhDYfgasaacH8akY=wiFfYdH8Gipec8Eeeu0xXdbba9frFj0=OqFfea0dXdd9vqai=hGuQ8kuc9pgc9s8qqaq=dirpe0xb9q8qiLsFr0=vr0=vr0dc8meaabaqaciaacaGaaeqabaqabeGadaaakeaadaaeWbqaaiabdMfaznaaBaaaleaacqWGQbGAaeqaaOGaeyypa0JaeGymaeJaeiOla4caleaacqWGQbGAcqGH9aqpcqaIXaqmaeaacqWGRbWAa0GaeyyeIuoaaaa@3948@

### Describing residues

As noted above, *X*_*i *_is a *description *of the residue at the *i*th position the current sequence window. We study two ways to describe residues.

The *amino acid identity *method sets *X*_*i *_to the name of the amino acid at position *i *in the sequence window. Thus, this method of description treats each *X*_*i *_as a variable with *nominal *values. For convenience, we let the names of the 20 amino acids plus the end-of-sequence marker be represented by the set *A *= {1, ..., 21}.

The PSI-BLAST *profile *method (first successfully applied by Jones [4]) requires that the target protein first be (multiply-) aligned with orthologous sequences. *X*_*i *_is set to the log-odds score vector (over the 20 possible amino acids character) derived from the multiple alignment column corresponding to position *i *in the window. This method of description treats each *X*_*i *_as a 21-dimensional vector of real values, the extra dimension being used to indicate if *X*_*i *_is off the end of the actual protein sequence (0 for within sequence, 0.5 for outside). The log-odds alignment scores are obtained by running PSI-BLAST against Genbank's standard non-redundant protein sequence database for three iterations. The elements in PSI-BLAST position-specific scoring matrices are divided by 10 so that most values appear between -1.0 and 1.0. The variation we use was successfully applied in the prediction of protein B-factor profiles [19].

### Models and algorithms

We mainly explore using two well-known machine learning methods: naive Bayes and probabilistic neural networks. We also develop and evaluate a cascaded variant of the probabilistic neural network (cf. the layered architecture in [15]). Finally, we construct a cascaded categorical neural network as a representative categorical model.

#### The Naive Bayes' Density Predictor

The classical Naive Bayes' algorithm assumes (naively) that the input features (*X*_*i*_, 1 ≤ *i *≤ *w*) are independent random variables given the class of the residue. Despite this simplifying assumption, NB classifiers often perform surprisingly well on empirical data [14]. The key step in the NB algorithm is to estimate the *class-conditional probability *of each feature given each class,

*p*_*ij*_(*x*) = *Pr*(*X*_*i *_= *x*|*C *= *j*), for 1 ≤ *i *≤ *w *and 1 ≤ *j *≤ *k*.

That is, *p*_*ij*_(*x*) is the probability that *X*_*i *_= *x *given that the class is *j*. The joint class-conditional probability is computed from these, using the assumption of independence, as

Pr(X|C=j)=∏i=1wPr(Xi|C=j).     (1)
 MathType@MTEF@5@5@+=feaafiart1ev1aaatCvAUfKttLearuWrP9MDH5MBPbIqV92AaeXatLxBI9gBaebbnrfifHhDYfgasaacH8akY=wiFfYdH8Gipec8Eeeu0xXdbba9frFj0=OqFfea0dXdd9vqai=hGuQ8kuc9pgc9s8qqaq=dirpe0xb9q8qiLsFr0=vr0=vr0dc8meaabaqaciaacaGaaeqabaqabeGadaaakeaaieGacqWFqbaucqWFYbGCcqGGOaakieqacqGFybawcqGG8baFcqWGdbWqcqGH9aqpcqWGQbGAcqGGPaqkcqGH9aqpdaqeWbqaaiab=bfaqjab=jhaYjabcIcaOiabdIfaynaaBaaaleaacqWGPbqAaeqaaOGaeiiFaWNaem4qamKaeyypa0JaemOAaOMaeiykaKIaeiOla4IaaCzcaiaaxMaadaqadaqaaiabigdaXaGaayjkaiaawMcaaaWcbaGaemyAaKMaeyypa0JaeGymaedabaGaem4DaChaniabg+Givdaaaa@4FC3@

The probabilities we are after-the posterior probabilities of the classes given the data- are gotten using Bayes' rule:

Pr(C=j|X)=Pr(X|C=j)Pr(C=j)Pr(X).
 MathType@MTEF@5@5@+=feaafiart1ev1aaatCvAUfKttLearuWrP9MDH5MBPbIqV92AaeXatLxBI9gBaebbnrfifHhDYfgasaacH8akY=wiFfYdH8Gipec8Eeeu0xXdbba9frFj0=OqFfea0dXdd9vqai=hGuQ8kuc9pgc9s8qqaq=dirpe0xb9q8qiLsFr0=vr0=vr0dc8meaabaqaciaacaGaaeqabaqabeGadaaakeaaieGacqWFqbaucqWFYbGCcqGGOaakcqWGdbWqcqGH9aqpcqWGQbGAcqGG8baFieqacqGFybawcqGGPaqkcqGH9aqpdaWcaaqaaiab=bfaqjab=jhaYjabcIcaOiabhIfayjabcYha8jabdoeadjabg2da9iabdQgaQjabcMcaPiab=bfaqjab=jhaYjabcIcaOiabdoeadjabg2da9iabdQgaQjabcMcaPaqaaiab=bfaqjab=jhaYjabcIcaOiab+HfayjabcMcaPaaacqGGUaGlaaa@50B6@

NB classifiers are usually trained using examples of **X **labelled with a *single class*. We use an extension of the algorithm that allows training using examples labelled with probability vectors, **T**, describing the posterior probabilities. We call this model a *Naive Bayes' Density Predictor *(NBDP). As mentioned above, we use two methods for describing the input sequences. We will now describe how we estimate the class-conditional probabilities for these two different input feature encoding methods.

With the amino acid identity method, the *X*_*i *_are nominal random variables that take 21 possible values, *x *∈ {1, 2, ..., 21}. Let *X*_*i*_(*E*) and *T*_*j*_(*E*) be the values of variables *X*_*i *_and *T*_*j *_for training example *E *=*<***X, T ***>*. The class-conditional probability for each combination of *i*, *j*, and *x *is estimated using the weighted maximum likelihood estimate,

p^ij(x)=∑{E∈S|Xi(E)=x}Tj(E)/∑E∈STj(E).
 MathType@MTEF@5@5@+=feaafiart1ev1aaatCvAUfKttLearuWrP9MDH5MBPbIqV92AaeXatLxBI9gBaebbnrfifHhDYfgasaacH8akY=wiFfYdH8Gipec8Eeeu0xXdbba9frFj0=OqFfea0dXdd9vqai=hGuQ8kuc9pgc9s8qqaq=dirpe0xb9q8qiLsFr0=vr0=vr0dc8meaabaqaciaacaGaaeqabaqabeGadaaakeaacuWGWbaCgaqcamaaBaaaleaacqWGPbqAcqWGQbGAaeqaaOGaeiikaGIaemiEaGNaeiykaKIaeyypa0ZaaabuaeaacqWGubavdaWgaaWcbaGaemOAaOgabeaakiabcIcaOiabdweafjabcMcaPiabc+caVaWcbaGaei4EaSNaemyrauKaeyicI4Saem4uamLaeiiFaWNaemiwaG1aaSbaaWqaaiabdMgaPbqabaWccqGGOaakcqWGfbqrcqGGPaqkcqGH9aqpcqWG4baEcqGG9bqFaeqaniabggHiLdGcdaaeqbqaaiabdsfaunaaBaaaleaacqWGQbGAaeqaaOGaeiikaGIaemyrauKaeiykaKcaleaacqWGfbqrcqGHiiIZcqWGtbWuaeqaniabggHiLdGccqGGUaGlaaa@5A96@

With PSI-BLAST profile method for encoding sequences, the *X*_*i *_are themselves vectors of *continuous *random variables. In particular, each feature vector, *X*_*i*_, is a vector of 21 real-valued *sub-features*. That is, *X*_*i *_=<*v*_1_, *v*_2_, ..., *v*_21 _>. We make a further (naive) assumption of independence: all of the 21·*w *sub-feature random variables are independent given the class. This allows us to simply expand the product in Equation 1 to be over *all of the components of all the vectors in ***X**. We then make an assumption that is commonly used with naive Bayes' classifiers when the input features are continuous: that each sub-feature, *X*, is a Gaussian random variable when conditioned on the class [20]. That is,

*Pr*(*X *= *x*|*C *= *j*) = *g*(*x*, *μ*, *σ*), 0 ≤ *j *≤ *k*,

where *g*() is the Gaussian density function. We estimate the mean, *μ *and standard deviation, *σ*, from the training set data using the weighted maximum likelihood estimates

μ^=∑E∈SX(E)Tj(E)|S|,     (2)
 MathType@MTEF@5@5@+=feaafiart1ev1aaatCvAUfKttLearuWrP9MDH5MBPbIqV92AaeXatLxBI9gBaebbnrfifHhDYfgasaacH8akY=wiFfYdH8Gipec8Eeeu0xXdbba9frFj0=OqFfea0dXdd9vqai=hGuQ8kuc9pgc9s8qqaq=dirpe0xb9q8qiLsFr0=vr0=vr0dc8meaabaqaciaacaGaaeqabaqabeGadaaakeaaiiGacuWF8oqBgaqcaiabg2da9maaqafabaWaaSaaaeaacqWGybawcqGGOaakcqWGfbqrcqGGPaqkcqWGubavdaWgaaWcbaGaemOAaOgabeaakiabcIcaOiabdweafjabcMcaPaqaaiabcYha8jabdofatjabcYha8baaaSqaaiabdweafjabgIGiolabdofatbqab0GaeyyeIuoakiabcYcaSiaaxMaacaWLjaWaaeWaaeaacqaIYaGmaiaawIcacaGLPaaaaaa@47D7@

and

σ^2=∑E∈S(X(E)Tj(E)−μ^)2|S|−1.     (3)
 MathType@MTEF@5@5@+=feaafiart1ev1aaatCvAUfKttLearuWrP9MDH5MBPbIqV92AaeXatLxBI9gBaebbnrfifHhDYfgasaacH8akY=wiFfYdH8Gipec8Eeeu0xXdbba9frFj0=OqFfea0dXdd9vqai=hGuQ8kuc9pgc9s8qqaq=dirpe0xb9q8qiLsFr0=vr0=vr0dc8meaabaqaciaacaGaaeqabaqabeGadaaakeaaiiGacuWFdpWCgaqcamaaCaaaleqabaGaeGOmaidaaOGaeyypa0ZaaabuaeaadaWcaaqaaiabcIcaOiabdIfayjabcIcaOiabdweafjabcMcaPiabdsfaunaaBaaaleaacqWGQbGAaeqaaOGaeiikaGIaemyrauKaeiykaKIaeyOeI0Iaf8hVd0MbaKaacqGGPaqkdaahaaWcbeqaaiabikdaYaaaaOqaaiabcYha8jabdofatjabcYha8jabgkHiTiabigdaXaaacqGGUaGlcaWLjaGaaCzcamaabmaabaGaeG4mamdacaGLOaGaayzkaaaaleaacqWGfbqrcqGHiiIZcqWGtbWuaeqaniabggHiLdaaaa@506F@

where *X*(*E*) is defined analogously to *X*_*i*_(*E*), above.

#### Probabilistic neural networks

We also explore the use of Probabilistic Neural Networks for which weight parameters, *W*, are adapted to produce probability distributions in accordance with the observed data [21]. We use a network with at most one hidden layer. We ask, what is the most likely explanation (in terms of our representation *W*) of our training set data *S*? That is, we maximise

Pr(W|S)=Pr(S|W)Pr(W)Pr(S)
 MathType@MTEF@5@5@+=feaafiart1ev1aaatCvAUfKttLearuWrP9MDH5MBPbIqV92AaeXatLxBI9gBaebbnrfifHhDYfgasaacH8akY=wiFfYdH8Gipec8Eeeu0xXdbba9frFj0=OqFfea0dXdd9vqai=hGuQ8kuc9pgc9s8qqaq=dirpe0xb9q8qiLsFr0=vr0=vr0dc8meaabaqaciaacaGaaeqabaqabeGadaaakeaaieGacqWFqbaucqWFYbGCcqGGOaakcqWGxbWvcqGG8baFcqWGtbWucqGGPaqkcqGH9aqpdaWcaaqaaiab=bfaqjab=jhaYjabcIcaOiabdofatjabcYha8jabdEfaxjabcMcaPiab=bfaqjab=jhaYjabcIcaOiabdEfaxjabcMcaPaqaaiab=bfaqjab=jhaYjabcIcaOiabdofatjabcMcaPaaaaaa@48FF@

(maximum *a posteriori*). More specifically, since *Pr*(*W*) is the same for all configurations (all weights are initialised using a zero-mean Gaussian) and since *Pr*(*S*) does not depend on *W*, we maximise only the likelihood *Pr*(*S*|*W*). The optimisation is standardly implemented by gradient descent on the relative cross entropy [21].

−∑E∈S∑j=1kTj(E)ln⁡Yj(E)
 MathType@MTEF@5@5@+=feaafiart1ev1aaatCvAUfKttLearuWrP9MDH5MBPbIqV92AaeXatLxBI9gBaebbnrfifHhDYfgasaacH8akY=wiFfYdH8Gipec8Eeeu0xXdbba9frFj0=OqFfea0dXdd9vqai=hGuQ8kuc9pgc9s8qqaq=dirpe0xb9q8qiLsFr0=vr0=vr0dc8meaabaqaciaacaGaaeqabaqabeGadaaakeaacqGHsisldaaeqbqaamaaqahabaGaemivaq1aaSbaaSqaaiabdQgaQbqabaGccqGGOaakcqWGfbqrcqGGPaqkaSqaaiabdQgaQjabg2da9iabigdaXaqaaiabdUgaRbqdcqGHris5aOGagiiBaWMaeiOBa4MaemywaK1aaSbaaSqaaiabdQgaQbqabaGccqGGOaakcqWGfbqrcqGGPaqkaSqaaiabdweafjabgIGiolabdofatbqab0GaeyyeIuoaaaa@4862@

The softmax output function is used, ensuring that all outputs sum up to 1. Neural networks require a lengthy training period and the setting of a learning rate. In preliminary trials we noted that the presentation of 20 000 sequences was sufficient to ensure convergence to a low training error. To achieve a stable descent on the error surface the learning rate was set to 0.001 (when the rate was 0.01 fluctuations occurred).

With the amino acid identity description method, feature *X*_*i *_= *k *is encoded as a 21-dimensional vector, **V **=<*v*_1_, *v*_2_, ..., *v*_21 _>, with *v*_*k *_= 1, and *v*_*j *_= 0 for *j *≠ *k*. For the PSI-BLAST profile description method, feature *X*_*i *_=<*v*_1_, *v*_2_, ..., *v*_21 _> is already encoded as a 21-dimensional real-valued vector. For each training example, **X**, the *w *encoded feature vectors are concatenated to create a single input vector of length 21·*w*.

#### Cascaded probabilistic neural networks

**Figure 3:**
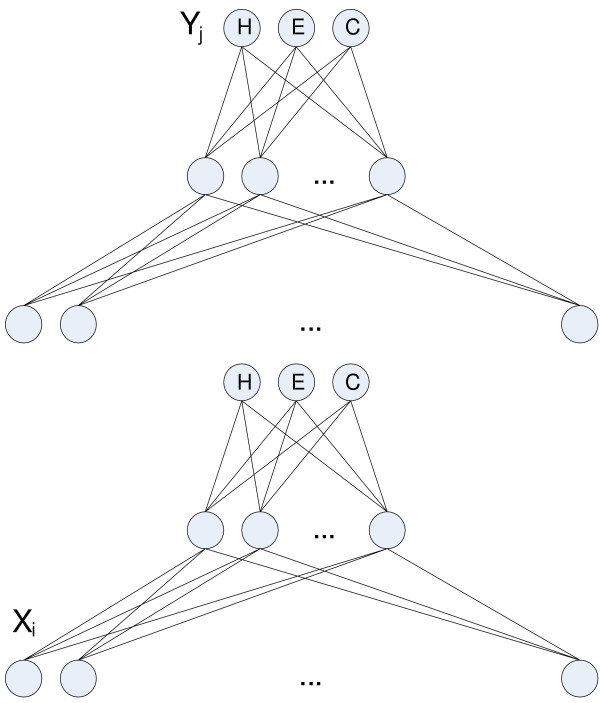
The architecture of the Cascaded Probabilistic Neural Network.

Similar to many successful categorical secondary structure predictors [15,4] we here investigate a layered architecture consisting of two coupled probabilistic neural networks (see Figure 3). The first is a sequence-to-structure network (a PNN as described above), the second is a structure-to-structure network, using consecutive predictions from the first-level network to predict the structure of the middle residue. We use the best PNN as our first-level network. New second-level networks are trained after the first-level networks and using the same learning method and parameters.

#### Categorical models: Cascaded categorical neural network

To put our work in a broader context, we explore the Cascaded Categorical Neural Network, a model representing the state-of-the-art of secondary structure classification [4]. The CCNN is essentially the same neural network model as employed in PSIPRED [4]. Moreover, with the exception of transformation of training targets and model outputs as explained below, the CCNN is identical to the CPNN.

The probabilistic target data is converted to categorical targets by choosing the majority class (the class with the highest probability),

class(T)=arg⁡max⁡j Tj.
 MathType@MTEF@5@5@+=feaafiart1ev1aaatCvAUfKttLearuWrP9MDH5MBPbIqV92AaeXatLxBI9gBaebbnrfifHhDYfgasaacH8akY=wiFfYdH8Gipec8Eeeu0xXdbba9frFj0=OqFfea0dXdd9vqai=hGuQ8kuc9pgc9s8qqaq=dirpe0xb9q8qiLsFr0=vr0=vr0dc8meaabaqaciaacaGaaeqabaqabeGadaaakeaacqWGJbWycqWGSbaBcqWGHbqycqWGZbWCcqWGZbWCcqGGOaakieqacqWFubavcqGGPaqkcqGH9aqpdaWfqaqaaiGbcggaHjabckhaYjabcEgaNjGbc2gaTjabcggaHjabcIha4bWcbaGaemOAaOgabeaakiabbccaGiabdsfaunaaBaaaleaacqWGQbGAaeqaaOGaeiOla4caaa@45B7@

The categorical target data is used to train CCNNs.

In order to compare our probalistic models with categorical prediction methods, we convert their outputs to categorical predictions. The probabilities predicted by the probabilistic models and the CCNN (fitted with the softmax output function) are converted by assigning the class corresponding to the output with the highest activity,

class(Y)=arg⁡max⁡j Yj.
 MathType@MTEF@5@5@+=feaafiart1ev1aaatCvAUfKttLearuWrP9MDH5MBPbIqV92AaeXatLxBI9gBaebbnrfifHhDYfgasaacH8akY=wiFfYdH8Gipec8Eeeu0xXdbba9frFj0=OqFfea0dXdd9vqai=hGuQ8kuc9pgc9s8qqaq=dirpe0xb9q8qiLsFr0=vr0=vr0dc8meaabaqaciaacaGaaeqabaqabeGadaaakeaacqWGJbWycqWGSbaBcqWGHbqycqWGZbWCcqWGZbWCcqGGOaakieqacqWFzbqwcqGGPaqkcqGH9aqpdaWfqaqaaiGbcggaHjabckhaYjabcEgaNjGbc2gaTjabcggaHjabcIha4bWcbaGaemOAaOgabeaakiabbccaGiabdMfaznaaBaaaleaacqWGQbGAaeqaaOGaeiOla4caaa@45CB@

### Training and testing the models

For training our models, we created a non-redundant dataset of continuum secondary structure data for 174 protein chains (set-174). This dataset was derived from a dataset used by Anderson *et al*. [2] for studying protein continuum secondary structures. All model design and testing of different model parameters described in this manuscript was done using only this dataset.

During model development, we used cross-validation on the training set to measure their predictive accuracy. After all model development was complete, we used two additional datasets for independent validation of the various models. We used sequences in CAFASP3 [17] to evaluate *categorical *prediction accuracy. Although none of the sequences in the CAFASP3 set are included in our training set, because it is already relatively small, we chose not to attempt to remove any sequences that might be homologous with our training set. Instead, we developed an independent set of continuum secondary structures for 286 sequences (set-286) for evaluation of the accuracy of the models in both the probabilistic and categorical prediction tasks.

Our training dataset (set-174) was derived from Anderson and colleagues [2] dataset containing 210 structurally non-homologous protein chains representing different families defined by FSSP [22]. The continuum secondary structure data in this dataset came from a selection of higher quality NMR protein chains, and at was based on at least 10 NMR models for each chain. We applied Hobohm and Sanders' redundancy reduction method [23] to select the largest set of representative chains with sequence identities less than 25% according to CLUSTALW [24]. This resulted in a dataset containing continuum secondary structure data for 174 protein chains containing approximately 17 thousand residues. This dataset was further divided into ten random groups with equal number of protein chains for cross-validation tests.

We created a test dataset (set-286) designed to have very low homology with our training set and very low redundancy within itself. We based the test dataset on all the NMR protein structures in the Protein Data Bank [25] published since 2003, after the publication date of the Anderson and colleagues dataset. To insure low homology with the training set, we removed all sequences from the test dataset with greater than 25% sequence identity with any sequence in the training set. To reduce the within-set redundancy, we then used Hobohm and Sanders method to select the largest subset of sequences with pairwise identity no more than 25%. We also removed all sequences shorter than 50 amino acids. These steps yielded a test dataset comprising 286 chains containing a total of 30214 residues. Their continuum secondary structures were obtained from the DSSPcont website [26].

All models (including both probabilistic and categorical models) are evaluated in two ways. Firstly, we perform 10-fold cross-validation on the training set (set-174). Secondly, we measure the accuracy on the sequences in the test dataset (set-286).

We use the Kullback-Leibler (KL) divergence [27,28] to measure the accuracy of our continuum secondary structure predictions. The KL divergence is the standard means of measuring the distance between two probability distributions, and is defined as

KL=∑j=1kTjlog⁡(Tj/Yj),
 MathType@MTEF@5@5@+=feaafiart1ev1aaatCvAUfKttLearuWrP9MDH5MBPbIqV92AaeXatLxBI9gBaebbnrfifHhDYfgasaacH8akY=wiFfYdH8Gipec8Eeeu0xXdbba9frFj0=OqFfea0dXdd9vqai=hGuQ8kuc9pgc9s8qqaq=dirpe0xb9q8qiLsFr0=vr0=vr0dc8meaabaqaciaacaGaaeqabaqabeGadaaakeaacqqGlbWscqqGmbatcqGH9aqpdaaeWbqaaiabdsfaunaaBaaaleaacqWGQbGAaeqaaaqaaiabdQgaQjabg2da9iabigdaXaqaaiabdUgaRbqdcqGHris5aOGagiiBaWMaei4Ba8Maei4zaCMaeiikaGIaemivaq1aaSbaaSqaaiabdQgaQbqabaGccqGGVaWlcqWGzbqwdaWgaaWcbaGaemOAaOgabeaakiabcMcaPiabcYcaSaaa@46C3@

where *k *is the number of classes to which an input can belong, **T **is the target probability vector, and **Y **is the predicted probability vector. A KL divergence value of 0 indicates perfect agreement between the two distributions, larger values indicate more divergence between them.

To evaluate the performance of the categorical versions of each of our models, we use several distinct accuracy metrics: *Q*_*k*_, correlation coefficient and SOV. Each of these metrics is based on counting the numbers of times a sample of a known class is assigned to the correct or incorrect class. We use the quantities *true positives*, *tp*(*C*), which is the number of test samples in class *C *predicted to be in class *C*, *true negatives*, *tn*(*C*), which is the number of test samples not in class *C *predicted not to be in class *C*, *false negatives*, *fn*(*C*), which is the number of test samples in class *C *predicted not to be in class *C*, and *false positives*, *fp*(*C*), which is the number of test samples not in class *C *predicted to be in class *C*. The *Q*_*k *_metric defines the accuracy of a *k*-class model as

Qk=∑j=1ktp(j)∑j=1ktp(j)+fn(j)⋅100.
 MathType@MTEF@5@5@+=feaafiart1ev1aaatCvAUfKttLearuWrP9MDH5MBPbIqV92AaeXatLxBI9gBaebbnrfifHhDYfgasaacH8akY=wiFfYdH8Gipec8Eeeu0xXdbba9frFj0=OqFfea0dXdd9vqai=hGuQ8kuc9pgc9s8qqaq=dirpe0xb9q8qiLsFr0=vr0=vr0dc8meaabaqaciaacaGaaeqabaqabeGadaaakeaacqWGrbqudaWgaaWcbaGaem4AaSgabeaakiabg2da9maalaaabaWaaabmaeaacqWG0baDcqWGWbaCcqGGOaakcqWGQbGAcqGGPaqkaSqaaiabdQgaQjabg2da9iabigdaXaqaaiabdUgaRbqdcqGHris5aaGcbaWaaabmaeaacqWG0baDcqWGWbaCcqGGOaakcqWGQbGAcqGGPaqkaSqaaiabdQgaQjabg2da9iabigdaXaqaaiabdUgaRbqdcqGHris5aOGaey4kaSIaemOzayMaemOBa4MaeiikaGIaemOAaOMaeiykaKcaaiabgwSixlabigdaXiabicdaWiabicdaWiabc6caUaaa@5675@

The Matthews correlation coefficient is defined as

r(C)=tp(C)⋅tn(C)−fp(C)⋅fn(C)(tp(C)+fn(C))(tp(C)+fp(C))(tn(C)+fp(C))(tn(C)+fn(C)).     (4)
 MathType@MTEF@5@5@+=feaafiart1ev1aaatCvAUfKttLearuWrP9MDH5MBPbIqV92AaeXatLxBI9gBaebbnrfifHhDYfgasaacH8akY=wiFfYdH8Gipec8Eeeu0xXdbba9frFj0=OqFfea0dXdd9vqai=hGuQ8kuc9pgc9s8qqaq=dirpe0xb9q8qiLsFr0=vr0=vr0dc8meaabaqaciaacaGaaeqabaqabeGadaaakeaacqWGYbGCcqGGOaakcqWGdbWqcqGGPaqkcqGH9aqpdaWcaaqaaiabdsha0jabdchaWjabcIcaOiabdoeadjabcMcaPiabgwSixlabdsha0jabd6gaUjabcIcaOiabdoeadjabcMcaPiabgkHiTiabdAgaMjabdchaWjabcIcaOiabdoeadjabcMcaPiabgwSixlabdAgaMjabd6gaUjabcIcaOiabdoeadjabcMcaPaqaamaakaaabaGaeiikaGIaemiDaqNaemiCaaNaeiikaGIaem4qamKaeiykaKIaey4kaSIaemOzayMaemOBa4MaeiikaGIaem4qamKaeiykaKIaeiykaKIaeiikaGIaemiDaqNaemiCaaNaeiikaGIaem4qamKaeiykaKIaey4kaSIaemOzayMaemiCaaNaeiikaGIaem4qamKaeiykaKIaeiykaKIaeiikaGIaemiDaqNaemOBa4MaeiikaGIaem4qamKaeiykaKIaey4kaSIaemOzayMaemiCaaNaeiikaGIaem4qamKaeiykaKIaeiykaKIaeiikaGIaemiDaqNaemOBa4MaeiikaGIaem4qamKaeiykaKIaey4kaSIaemOzayMaemOBa4MaeiikaGIaem4qamKaeiykaKIaeiykaKcaleqaaaaakiabc6caUiaaxMaacaWLjaWaaeWaaeaacqaI0aanaiaawIcacaGLPaaaaaa@8911@

Finally, we also illustrate SOV [16], a segment-based, standard measure of secondary structure prediction accuracy that is designed to capture the "usefulness" of the predictions. We used software provided by the authors to compute SOV, and refer the reader to the paper for details of its definition.

For cross-validation, the training dataset is divided randomly into ten roughly equal-sized subsets, each subset appearing as test subset in exactly once of ten separate training sessions (ensuring that each sample appears as a test case exactly once). For each run of cross-validation, we compute the mean KL divergence (averaged over each residue in the sequences in the test subsets) and a single value for each categorical accuracy metric. In order to measure the standard error of the categorical metrics, we average their values over five independent cross-validation runs. Standard error is the sample standard deviation divided by the square root of the number of samples (five in this case).

Predictive accuracy on the test dataset (set-286) is measured using the models created and trained using the *training *dataset during the cross-validation runs. Each categorical accuracy measure is computed independently for each model and then averaged. We report the mean KL divergence, averaged over all residues in the test dataset. The KL divergence for a single residue is computed by first averaging the predictions of all models (of a given type) for the residue, and computing the KL divergence of the average prediction and the target density.

We define the target entropy as

∑j=1k−Tjlog⁡k(Tj).
 MathType@MTEF@5@5@+=feaafiart1ev1aaatCvAUfKttLearuWrP9MDH5MBPbIqV92AaeXatLxBI9gBaebbnrfifHhDYfgasaacH8akY=wiFfYdH8Gipec8Eeeu0xXdbba9frFj0=OqFfea0dXdd9vqai=hGuQ8kuc9pgc9s8qqaq=dirpe0xb9q8qiLsFr0=vr0=vr0dc8meaabaqaciaacaGaaeqabaqabeGadaaakeaadaaeWbqaaiabgkHiTiabdsfaunaaBaaaleaacqWGQbGAaeqaaaqaaiabdQgaQjabg2da9iabigdaXaqaaiabdUgaRbqdcqGHris5aOGagiiBaWMaei4Ba8Maei4zaC2aaSbaaSqaaiabdUgaRbqabaGccqGGOaakcqWGubavdaWgaaWcbaGaemOAaOgabeaakiabcMcaPiabc6caUaaa@4253@

Similarly, the predicted entropy is based on the model's output

∑j=1k−Yjlog⁡k(Yj).
 MathType@MTEF@5@5@+=feaafiart1ev1aaatCvAUfKttLearuWrP9MDH5MBPbIqV92AaeXatLxBI9gBaebbnrfifHhDYfgasaacH8akY=wiFfYdH8Gipec8Eeeu0xXdbba9frFj0=OqFfea0dXdd9vqai=hGuQ8kuc9pgc9s8qqaq=dirpe0xb9q8qiLsFr0=vr0=vr0dc8meaabaqaciaacaGaaeqabaqabeGadaaakeaadaaeWbqaaiabgkHiTiabdMfaznaaBaaaleaacqWGQbGAaeqaaaqaaiabdQgaQjabg2da9iabigdaXaqaaiabdUgaRbqdcqGHris5aOGagiiBaWMaei4Ba8Maei4zaC2aaSbaaSqaaiabdUgaRbqabaGccqGGOaakcqWGzbqwdaWgaaWcbaGaemOAaOgabeaakiabcMcaPiabc6caUaaa@4267@

High entropy (max is 1) means high variability.

## Authors' contributions

MB designed, programmed, performed and analysed most simulations, as well as drafted an early version of the manuscript. ZY initiated the project and participated in the design of the project and prepared the datasets. TLB contributed with technical and methodological ideas, developed the NBDP, and wrote and revised significant portions of the manuscript. All authors read and approved the final manuscript.

## References

[B1] Kabsch W, Sander C (1983). Dictionary of protein secondary structure: Pattern recognition of hydrogen bonded and geometrical features. Biopolymers.

[B2] Andersen CAF, Palmer AG, Brunak S, Rost B (2002). Continuum secondary structure captures protein flexibility. Structure.

[B3] Carter P, Andersen CAF, Rost B (2003). DSSPcont: continuous secondary structure assignments for proteins. Nucleic Acids Research.

[B4] Jones DT (1999). Protein secondary structure prediction based on position-specific scoring matrices. Journal of Molecular Biology.

[B5] Pollastri G, Przybylski D, Rost B, Baldi P (2002). Improving the Prediction of Protein Secondary Strucure in Three and Eight Classes Using Recurrent Neural Networks and Profiles. Proteins: Structure, Function, and Genetics.

[B6] Nordahl-Petersen T, Lundegaard C, Nielsen M, Bohr H, Bohr J, Brunak S, Gippert GP, Lund O (2000). Prediction of protein secondary structure at 80% accuracy. Proteins: Structure, Function and Genetics.

[B7] Rost B (2001). Protein Secondary Structure Prediction Continues to Rise. Journal of Structural Biology.

[B8] Hua S, Sun Z (2001). A novel method of protein secondary structure prediction with high segment overlap measure: support vector machine approach. Journal of Molecular Biology.

[B9] Ward JJ, McGuffin LJ, Buxton BF, Jones DT (2003). Secondary structure prediction with support vector machines. Bioinformatics.

[B10] Solis AD, Rackovsky S (2004). On the use of secondary structure in protein structure prediction: a bioinformatic analysis. Polymer.

[B11] Guermeur Y, Pollastri G, Elisseeff A, Zelus D, Paugam-Moisy H, Baldi P (2004). Combining protein secondary structure prediction models with ensemble methods of optimal complexity. Neurocomputing.

[B12] Frishman D, Argos  P (1995). Knowledge-based protein secondary structure assignment. Proteins: Structure, Function, and Genetics.

[B13] Kihara D (2005). The effect of long-range interactions on the secondary structure formation of proteins. Protein Sci.

[B14] Domingos P, Pazzani M (1997). On the optimality of the simple Bayesian classifier under zero-one loss. Machine Learning.

[B15] Rost B, Sander C (1993). Prediction of Protein Secondary Structure at Better than 70% Accuracy. Journal of Molecular Biology.

[B16] Zemla A, Venclovas C, Fidelis K, Rost B (1999). A modified definition of SOV, a segment-based measure for protein secondary structure prediction assessment. Proteins.

[B17] Eyrich VA, Przybylski D, Koh IYY, Grana O, Pazos F, Valencia A, Rost B (2003). CAFASP3 in the spotlight of EVA. Proteins: Structure, Function, and Genetics.

[B18] Young M, Kirshenbaum K, Dill K, Highsmith S (1999). Predicting conformational switches in proteins. Protein Sci.

[B19] Yuan Z, Bailey TL, Teasdale R (2005). Prediction of protein B-factor profiles. Proteins: Structure, Function, and Bioinformatics.

[B20] John GH, Langley P (1995). Estimating continuous distributions in Bayesian classifiers. Proceedings of the Eleventh Conference on Uncertainty in Artificial Intelligence.

[B21] Jordan MI, Bishop C, Tucker AB (1997). Neural networks. CRC Handbook of Computer Science.

[B22] Holm L, Sander C (1998). Touring protein fold space with Dali/FSSP. Nucleic Acids Research.

[B23] Hobohm U, Scharf M, Schneider R, Sander C (1992). Selection of representative protein data sets. Protein Science.

[B24] Thompson J, Higgins D, Gibson T (1994). CLUSTAL W: improving the sensitivity of progressive multiple sequence alignment through sequence weighting, position-specific gap penalties and weight matrix choice. Nucleic Acids Research.

[B25] Berman H, Westbrook J, Feng Z, Gilliland G, Bhat T, Weissig H, Shindyalov I, Bourne P (2000). The Protein Data Bank. Nucleic Acids Research.

[B26] DSSPcont. http://cubic.bioc.columbia.edu/services/DSSPcont.

[B27] Baldi P, Brunak S, Chauvin Y, Andersen CAF, Nielsen H (2000). Assessing the accuracy of prediction algorithms for classification: an overview. Bioinformatics.

[B28] Durbin R, Eddy SR, Krogh A, Mitchison G (1998). Biological Sequence Analysis.

